# *TaPP2C1*, a Group F2 Protein Phosphatase 2C Gene, Confers Resistance to Salt Stress in Transgenic Tobacco

**DOI:** 10.1371/journal.pone.0129589

**Published:** 2015-06-09

**Authors:** Wei Hu, Yan Yan, Xiaowan Hou, Yanzhen He, Yunxie Wei, Guangxiao Yang, Guangyuan He, Ming Peng

**Affiliations:** 1 Key Laboratory of Biology and Genetic Resources of Tropical Crops, Ministry of Agriculture, Institute of Tropical Bioscience and Biotechnology, Chinese Academy of Tropical Agricultural Sciences, Haikou, Hainan, 571101, China; 2 The Genetic Engineering International Cooperation Base of Chinese Ministry of Science and Technology, Key Laboratory of Molecular Biophysics of Chinese Ministry of Education, College of Life Science and Technology, Huazhong University of Science & Technology, Wuhan, 430074, China; University of Delhi South Campus, INDIA

## Abstract

Group A protein phosphatases 2Cs (PP2Cs) are essential components of abscisic acid (ABA) signaling in Arabidopsis; however, the function of group F2 subfamily PP2Cs is currently less known. In this study, TaPP2C1 which belongs to group F2 was isolated and characterized from wheat. Expression of the TaPP2C1-GFP fusion protein suggested its ubiquitous localization within a cell. *TaPP2C1* expression was downregulated by abscisic acid (ABA) and NaCl treatments, but upregulated by H_2_O_2_ treatment. Overexpression of *TaPP2C1 *in tobacco resulted in reduced ABA sensitivity and increased salt resistance of transgenic seedlings. Additionally, physiological analyses showed that improved resistance to salt stress conferred by *TaPP2C1 *is due to the reduced reactive oxygen species (ROS) accumulation, the improved antioxidant system, and the increased transcription of genes in the ABA-independent pathway. Finally, transgenic tobacco showed increased resistance to oxidative stress by maintaining a more effective antioxidant system. Taken together, these results demonstrated that TaPP2C1 negatively regulates ABA signaling, but positively regulates salt resistance. TaPP2C1 confers salt resistance through activating the antioxidant system and ABA-independent gene transcription process.

## Introduction

Abscisic acid (ABA) signaling plays critical roles in regulating plants’ responses to abiotic stress. When abiotic stress occurs, content of ABA dramatically increases in plant and promotes stomatal closure, antioxidative system, growth, and development, protecting vegetative tissues from injury [1]. Some studies have begun to clarify the ABA signaling pathway [2]. Several important components of ABA signaling, including ABA receptors PYR/PYL/RCARs, negative regulators PP2Cs, and positive regulators SnRK2s have been characterized in Arabidopsis. These components constitute a double negative modulatory model. PP2C inhibits SnRK2 through its direct dephosphorylation in the absence of ABA. In response to developmental or environmental clues, ABA signal induces the interaction between PYR/PYL/RCAR and PP2C, leading to PP2C inhibition and SnRK2 activation.

Protein phosphatases 2C (PP2Cs), a group of monomeric protein phosphatases, are found in humans, yeast, and plants [3]. To date, genome wide analyses have found 80 and 78 *PP2Cs* in *Arabidopsis thaliana* and *Oryza sativa* respectively. PP2Cs in Arabidopsis and rice have been further classified into 13 and 11 groups respectively, according to phylogenic analyses, gene structures, and protein motifs [4]. Accumulated evidence has confirmed that group A PP2Cs play negative roles in ABA signaling, of which some crucial members have been characterized, including AHG1, HAB1, HAB2, ABI1, ABI2 and PP2CA in *A*. *thaliana* [5–8]. These PP2Cs play both independent and overlapping roles in ABA signaling because double and triple mutants exhibit enhanced ABA sensitivity for stomatal movement, seed germination or gene transcription [9]. The exact roles of some group A PP2Cs in ABA signaling transduction were clarified by detailed genetic and biochemical analyses. PYR/PYL/RCAR can interact with ABI1, ABI2, HAB1, and AHG3 in the absence of ABA, which inhibits PP2C activity and activates downstream targets, such as SnRK2 and other PP2C substrates. This results in phosphorylation or activation of bZIP transcription factors ABF/AREB/ABI5, Slow Anion Channel SLAC1, and other ABA-responsive genes [2,10]. The ABA-induced PYL-PP2C interaction model in ABA signaling was further supported by *in vitro* reconstitution in *A*. *thaliana* protoplasts [11].

Due to the prominent role of ABA signaling in the abiotic stress response, some studies have also confirmed the function of PP2Cs in ABA-mediated abiotic stress resistance. For example, *AtPP2CA* was induced by ABA and abiotic stress, and transgenic antisense lines displayed improved resistance to freezing stress and increased sensitivity to ABA [12]. The interaction between ABI2 and salt overly sensitive 2 (SOS2) was destroyed by *abi2-1* mutation, which caused an improved salt stress resistance and ABA insensitivity in *A*. *thaliana* [13]. These results suggest that group A PP2C, PP2CA, and ABI2 are negative regulators of plant resistance to freezing, salt, and ABA signaling; however, overexpression of a group G *PP2C* gene, *AtPP2CG1*, increased salt resistance of *A*. *thaliana*. The expression levels of *AtPP2CG1* in the *abi2-3* were lower relative to wild type (WT) plants under salt treatment. These results demonstrate that *AtPP2CG1* positively modulate salt stress response with ABA-dependent manner [14], which suggests that, besides group A PP2Cs, other subfamilies of PP2Cs may participate in abiotic stress response and ABA signaling. Additionally, microarray analyses have identified 47 PP2Cs genes across all the 13 subgroups that respond to ABA and abiotic stress at transcriptional levels in *A*.*thaliana* [4]. However, there is less evidence of PP2Cs function in other subfamilies involved in ABA signaling and abiotic stress, especially for the group F2 PP2Cs.

Soil salinity severely limits growth and development of plants and leads to significant loss of crop yield [15]. Thus, investigation of mechanisms underlying the abiotic stress response is necessary to improve stress resistance in wheat. In this study, we characterized the function of the first wheat *PP2C* gene *TaPP2C1* (a group F2 subfamily PP2C), which confers resistance to salt stress by inducing the antioxidant system and ABA-independent gene expression system.

## Materials and Methods

### Wheat growth conditions and treatments

Wheat (*Triticum aestivum* L. cv. Chinese Spring) seeds were surface-sterilized with 75% (v/v) ethanol for 2 min and 1% (v/v) mercury chloride for 10 min and washed three times in sterile water. The wheat seedlings were cultured in a growth chamber at 200 μmol m^-2^s^-1^, 25°C, 16 h light photo-period. For NaCl treatment, the 10 d old wheat seedlings were transferred into 200 mM NaCl solution for 24 h. For signaling molecule treatments, the 10 d old seedlings were sprayed with 100 μM ethylene, 100 μM ABA, 100 μM methyl jasmonate (MeJA), 2 mM salicylic acid (SA), 50 μM auxin, or 10 mM H_2_O_2_ respectively and the seedlings were cultured for different time. For expression analysis of different organs, stems, roots, leaves, stamens, lemma and pistils were collected from wheat plants grown in a chamber. After frozen in liquid nitrogen, the samples were preserved at -70°C for RNA isolation and subsequent real-time quantitative polymerase chain reaction (qRT-PCR) analysis.

### Cloning of *TaPP2C1* gene

The wheat expressed sequence tag (TC382443) belonging to PP2C family was obtained from DFCI database. The 3’-ends of the gene was acquired with the RACE cDNA amplification kit (Clontech, USA) using the primer pairs P1 (S1 Table). The full-length cDNA sequence was amplified using primer pairs P2 (S1 Table) based on the indication of DNAMAN software. The PCR products were cloned and subjected to sequence analysis by BLAST.

### qRT-PCR

Expressions of *TaPP2C1* in wheat and stress/ABA-responsive genes in transgenic tobacco plants were determined with qRT-PCR method in MJ Research Opticon 2 instrument. Primers (P3-P14 in S1 Table) used in qRT-PCR detection had high specificity and efficiency according to agarose gel electrophoresis, melting curve and sequence analyses. Prior to actual experiments, several primer and template dilutions were performed to test the appropriate primer and template concentration. In all of the experiments, each sample contains four replicates and possible contamination was excluded by appropriate negative controls. *NtUbiquitin* and *TaActin* were used as reference gene for tobacco and wheat respectively to normalize the transcripts of target genes. The relative expression levels of the target genes were assessed according to the 2^-ΔΔCt^ method [16].

### Subcellular localization of TaPP2C1 protein

The ORF of *TaPP2C1* containing the *Xba*I restriction sites was obtained using primers (P15 in S1 Table), which was subsequently inserted into pBI121 to generate the TaPP2C1-GFP fusion protein under control of the CaMV 35S promoter. The pBI121-TaPP2C1-GFP and the pBI121-GFP were separately transformed into onion epidermal cells by gene gun mediated method. Fluorescence was examined by a microscopy (IX71, OLYMPUS, Japan). Additionally, the pBI121-TaPP2C1-GFP was also introduced into *Nicotina benthamiana* leaf cells using *Agrobacterium*-mediated transient expression method. GFP fluorescence was observed by confocal laser scanning microscopy (LSM710: Karl Zeiss, Jena, Germany).

### Generation of *TaPP2C1*-overexpressing transgenic tobacco lines

The pBI121-TaPP2C1-GFP and control pBI121-GFP constructs were transformed into tobaccos plants using *Agrobacterium*-mediated method as described by Horsch et al. (1985) [17]. The kanamycin-tolerant seedlings of T_1_ generation were further measured by PCR assay with primer pairs for both *GFP* (P16 in S1 Table) and *TaPP2C1* (P3 in S1 Table). Three homozygous T_3_ generation seedlings were used for further functional analysis. In this process, the vacant vector was also transformed into tobacco and served as a negative control. The transcriptional levels of *TaPP2C1* in the three homozygous T_3_ lines were determined by RT-PCR with primers P3 and P5 (S1 Table).

### ABA response and stress resistance analysis of transgenic lines

For ABA sensitivity assay, tobacco seeds were sown on MS or MS with 0.5 or 1 μM ABA for 12 days, and then the germination rates were calculated. In addition, 7 d old seedlings were transferred to MS or MS with 0.5 or 1μM ABA for 7 d, then the root length was measured, and then the ABA (1 μM) treated and control seedlings were collected to determine transcriptional levels of ABA associated genes. For salt stress resistance assay during germination, seeds from transgenic tobacco lines and control plants were sown on MS or MS with 200 mM NaCl for 8 d to test the germination rate. For salt resistance analysis in early seedlings, 7 d old tobacco seedlings from wild type (WT), vector control (VC) and transgenic plants were placed on MS or MS containing 100–200 mM NaCl for 7 d, then the root length was measured, and then the NaCl (200 mM) treated and control seedlings were sampled to test the transcriptional levels of ABA- and stress- associated genes. For salt stress resistance assay in adult seedlings, 21 d old seedlings were treated with 300 mM NaCl stress for 40 d, and then the survival rates were calculated. After 20 or 35 d of salt stress, the leaf samples were collected to determine the content of ion leakage (IL), Malondialdehyde (MDA), and H_2_O_2_, and the activities of anti-oxidative enzymes. Additionally, 42 d old seedlings were applied with 300 mM NaCl solution for 90 d, and then the photographs were taken. For oxidative stress resistance analysis, 7 d old seedlings were cultured on MS containing 30 μM methyl viologen (MV) for 10 d, and then the photos were taken. The whole seedlings were sampled to examine H_2_O_2_ content, activities and transcripts of SOD and CAT. Besides, 7 d old seedlings were incubated on MS with 5 mM H_2_O_2_ for 14 d, and then the photos were taken.

### Measurement of antioxidant enzyme activity, IL, MDA, and reactive oxygen species (ROS) accumulation

The activities of POD, SOD and CAT were detected using spectrophotometric method. Samples were ground to homogenate, which was subsequently homogenized in extraction buffer containing 1% polyvinylpyrrolidone and 0.05 M phosphate buffer (pH 7.8). After centrifugation, the resulting supernatant was used for the measurement of enzyme activities. Activities of CAT and SOD were detected with CAT and SOD Detection Kit (A007 and A001, Jiancheng, China). POD activity was examined using the method by Polle et al. (1994) [18]. Seven-day-old seedlings were cultured on MS containing 200 mM NaCl for 7 d to detect the H_2_O_2_ accumulation. Histochemical examination of H_2_O_2_ was conducted according to the method in previous study [19]. MDA content was detected by the colorimetric method according to Heath and Packer (1968) [20]. H_2_O_2_ content and IL were examined using the method by Jiang and Zhang (2001) [21].

## Results

### Cloning of *TaPP2C1* gene

The full-length cDNA, designated *TaPP2C1* (GenBank Accession: HQ287800), was isolated from wheat with RACE technique. *TaPP2C1* cDNA consists of 1138 bp with a 855 bp ORF endcoding the TaPP2C1 with 284 amino acid residues. Blastx-assisted sequence alignment suggested that *TaPP2C1* shares high sequence similarity with *PP2Cs* from other plant species: 97% sequence identity with *BdPP2C45* from *Brachypodium distachyon*, 92% with *OsPP2C45* from *O*. *sativa*, 90% with *ZmPP2C* from *Zea mays* and 76% with *AtPP2C59* from *A*. *thaliana*. Multiple alignment analysis indicated that TaPP2C1 has 11 conserved motifs characteristic of all Ser/Thr PP2Cs and 6 conserved residues that may be related to coordinating phosphate and metal ions (S1 Fig). Phylogenetic analysis of TaPP2C1 with *A*. *thaliana* PP2Cs showed that TaPP2C1 is grouped into F2 subfamily (S2 Fig). These results indicate that the *TaPP2C1* acquired in this study is a group F2 subfamily member of the *PP2Cs* in wheat.

### TaPP2C1 is localized throughout cells

**Fig 1:**
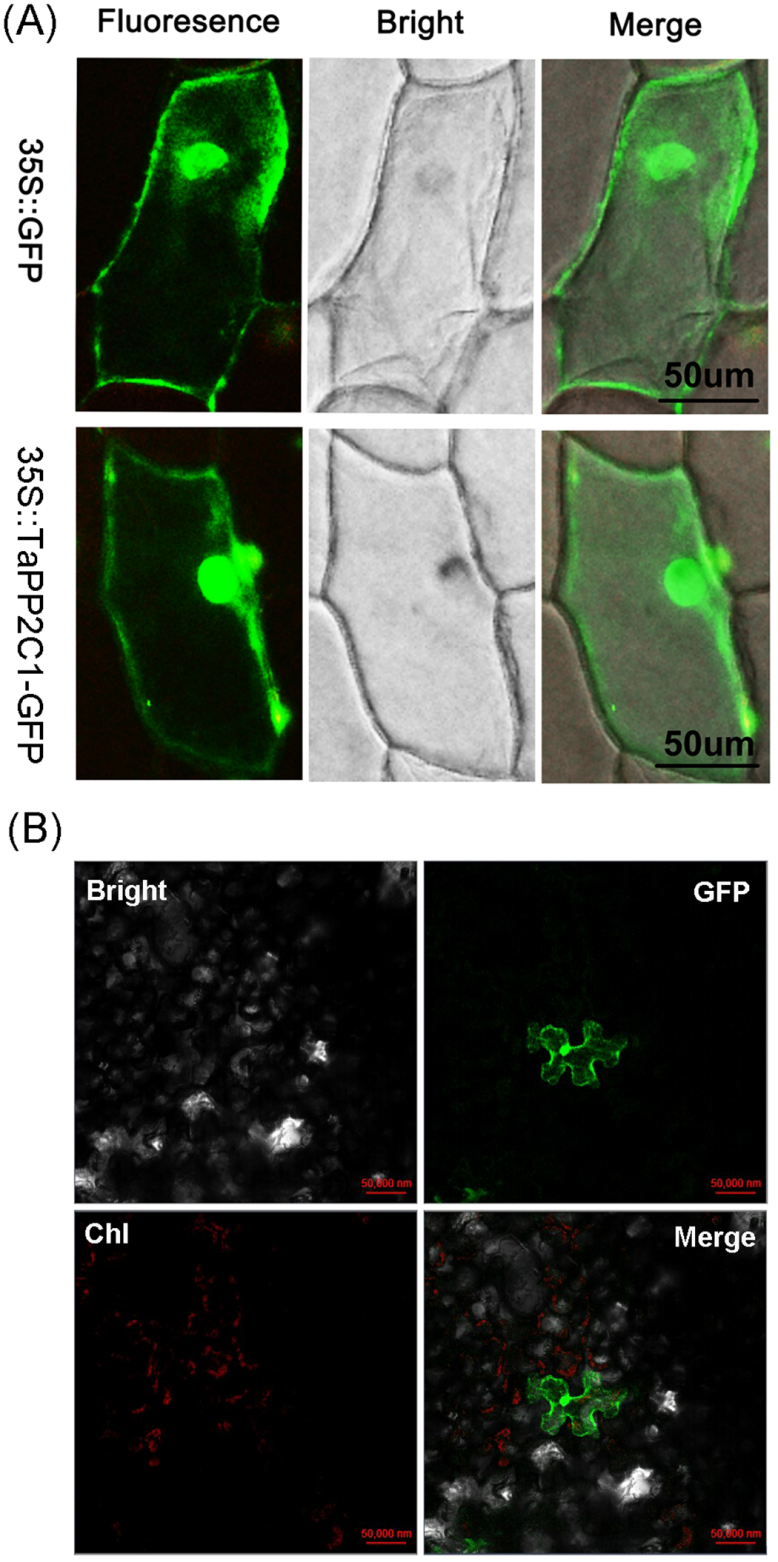
Subcellular localization of TaPP2C1 protein. (A) 35S::TaPP2C1-GFP and 35 S::GFP (positive control) constructs were transiently expressed in onion epidermal cells and observed with a microscopy after 48 h of bombardment. (B) 35S::TaPP2C1-GFP were transiently expressed in tobacco leaf cells. Two independent experiments produced similar results.

The subcellular location of the TaPP2C1 protein was investigated using transient expression assays with a 35S::TaPP2C1-GFP fusion protein in onion epidermal cell by particle bombardment. Fluorescence of the 35S::TaPP2C1-GFP fusion protein was associated with the nucleus, cytoplasm and plasma membrane of the onion epidermal cells (Fig 1A). The fluorescence of cells transformed with the 35S::GFP (positive control) was detectable throughout the cells (Fig 1A). In addition, the subcellular localization of TaPP2C1 was also determined in *Nicotina benthamiana* leaf cells using *Agrobacterium*-mediated transient expression system. The 35S::TaPP2C1-GFP fusion protein was found to be ubiquitously localized within a cell (Fig 1B). These results suggest that TaPP2C1 shows ubiquitous localization within a cell.

### *TaPP2C1* is downregulated by NaCl and ABA, but upregulated by H_2_O_2_ treatments

**Fig 2:**
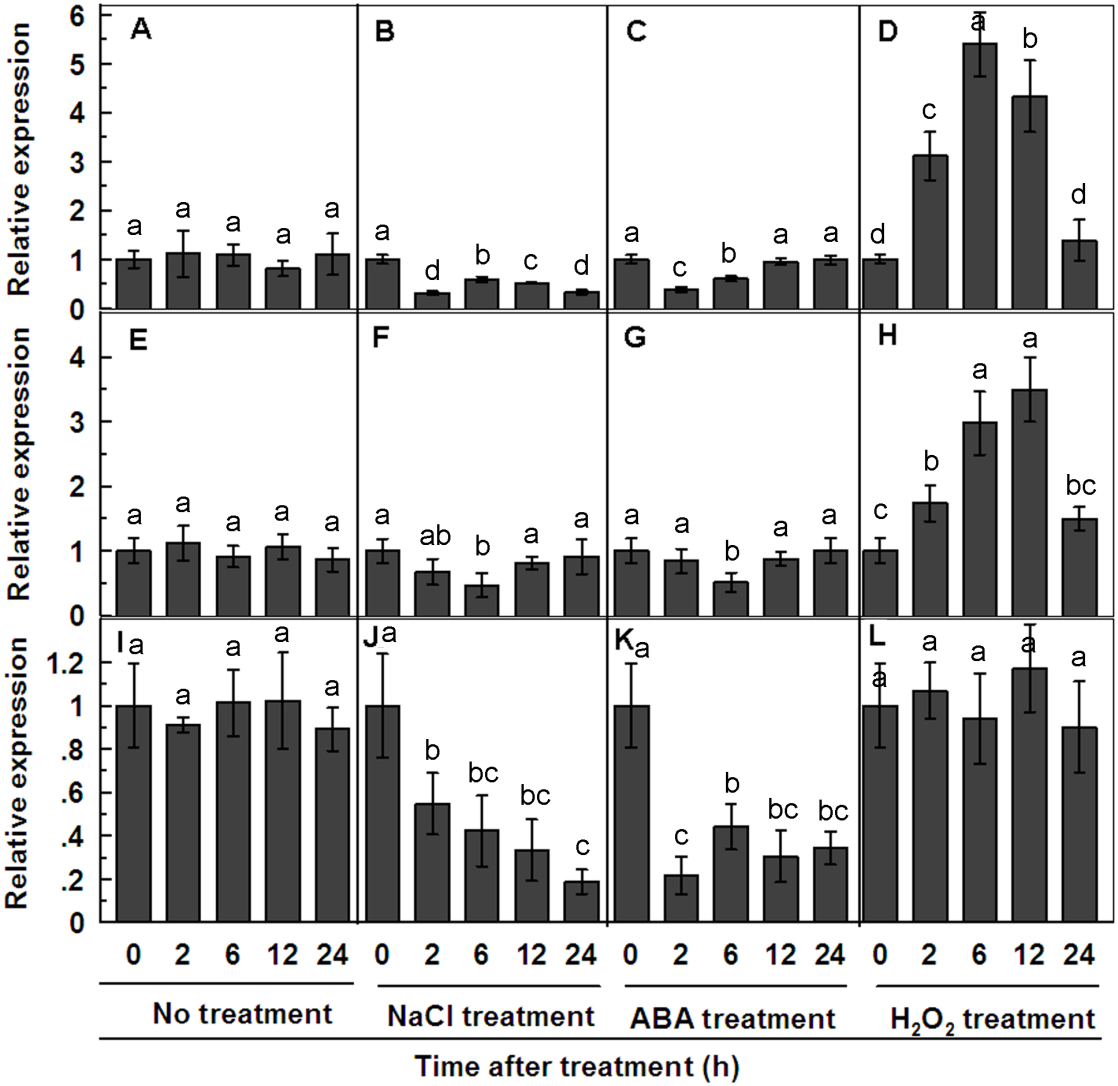
Expression profiles of *TaPP2C1* under standard condition and after NaCl, ABA, and H_2_O_2_ treatments in leaves (A-D), roots (E-H) and stems (I-L) of wheat by qRT-PCR analysis. The mRNA fold difference was relative to that of samples at 0 h. Data are means ±SD of n = 4 independent experiments. Means denoted by the same letter do not significantly differ at *P* <0.05 as determined by Duncan’s multiple range test.

*TaPP2C1* expression in different organs was examined using qRT-PCR, which indicated that *TaPP2C1* expressed in all tissues examined, and higher expression of the gene was detected in the stem and leaf (S3 Fig). To test changes in the expression of *TaPP2C1* responding to salt stress, the transcripts of *TaPP2C1* was detected under NaCl treatment. Results showed that the expression of *TaPP2C1* was downregulated in leaves and stems within 24 h of NaCl treatment and in roots at 6 h of NaCl treatment (Fig 2B, 2F and 2J). In addition, changes in the expression of *TaPP2C1* were also investigated after the treatments of multiple signals induced by salt stress. The results suggested that the *TaPP2C1* transcript was downregulated in stems within 24 h of ABA treatment, in leaves at 2 h and 6 h ABA treatment and in roots at 6 h ABA treatment (Fig 2C, 2G and 2K), but was upregulated in leaves and roots during 2–12 h of H_2_O_2_ treatment (Fig 2D and 2H). *TaPP2C1* transcript was also downregulated by MeJA within 24 h treatment and slightly upregulated by SA, ethylene, and auxin treatments at some time points in leaves (S4 Fig). Generally, these results indicate that the expression of *TaPP2C1* shows downregulation after NaCl, ABA and MeJA treatments, but upregulation by H_2_O_2_ treatment.

### Generation of the *TaPP2C1*-overexpressing transgenic tobacco lines

In order to further understand the function of *TaPP2C1 in planta*, *TaPP2C1-*overexpressing tobacco plants were generated under control of the constitutive promoter CaMV 35S. Three homozygous T_3_ lines were acquired and the transcriptional levels of *TaPP2C1* in the three transgenic lines were examined using RT-PCR assay (S5 Fig). The results indicate that *TaPP2C1* transcripts are present in transgenic lines but are not detected in the WT and VC, in which OE2 and OE8 have higher *TaPP2C1* expression.

### Overexpression of *TaPP2C1* in tobacco decreases plant sensitivity to ABA

**Fig 3:**
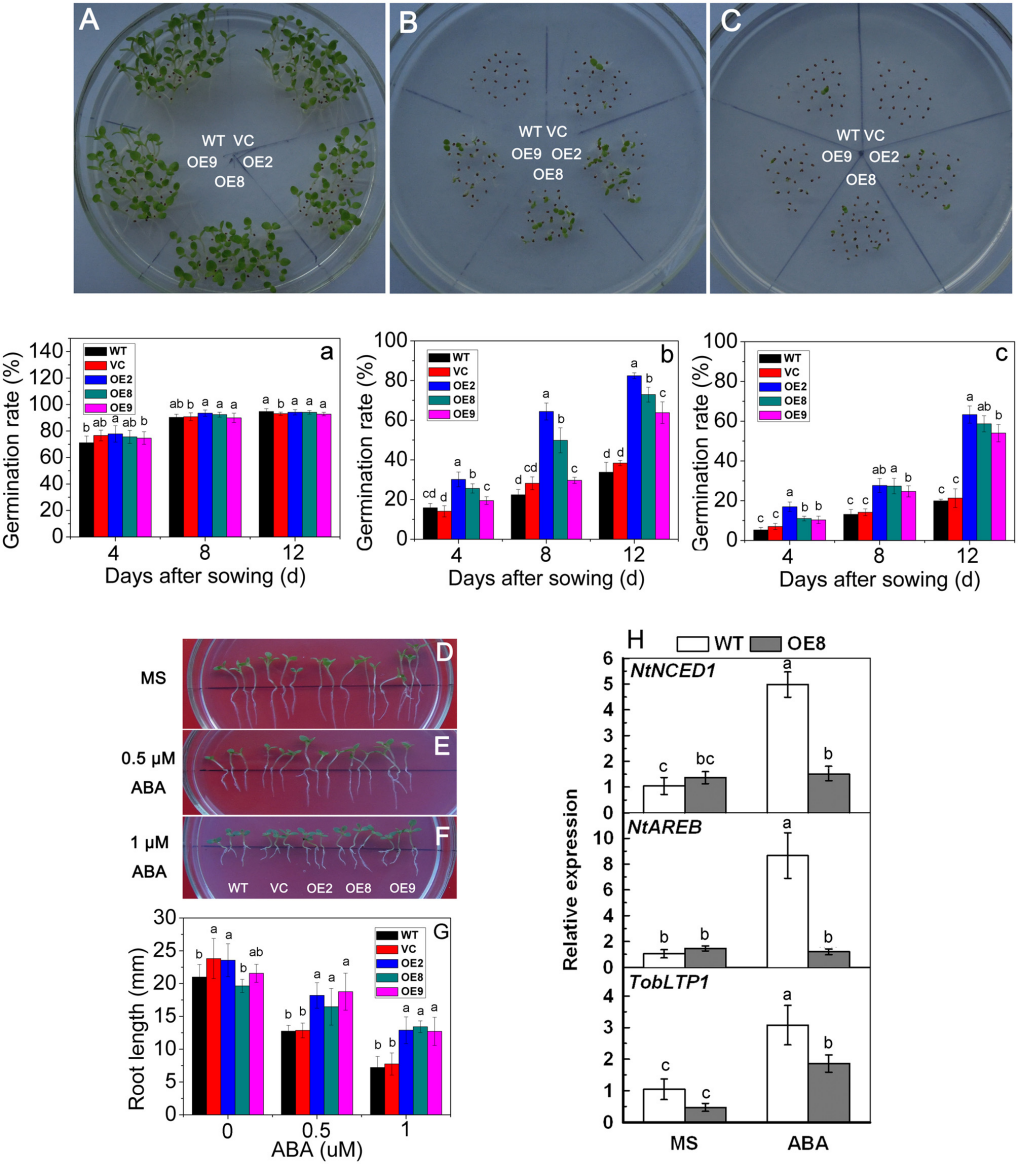
ABA sensitivity of *TaPP2C1*-overexpressing tobacco plants. Seedling phenotypes (A, B, C) and germination rates (a, b, c) of WT and transgenic lines under standard condition (A, a), 0.5 μM ABA (B, b), or 1 μM ABA (C, c) treatment. A, B and C are photos of the 12 d after germination on media. Seedling phenotypes (D, E, F) and root length (G) of WT and transgenic lines under standard condition (D), 0.5 μM ABA (E) or 1 μM ABA (F) treatment. Relative expression levels of ABA associated genes in transgenic line (OE 8) and WT under normal condition or 1μM ABA treatment (H). The mRNA fold difference was relative to that of WT samples under normal condition. Data are means ±SD of n = 4 independent experiments. Means denoted by the same letter do not significantly differ at *P* <0.05 as determined by Duncan’s multiple range test.

In order to examine whether *TaPP2C1* is involved in the ABA signaling, seed germination and primary root length were examined after ABA treatment. The seeds from controls and transgenic plants were directly sown on MS or ABA-containing MS medium for testing seed germination. Transgenic seeds showed similar germination rates to controls under normal condition (Fig 3A and 3a); however, control seeds had reduced germination rate after ABA treatment compared to the transgenic lines (Fig 3B, 3C, 3b and 3c). Meanwhile, primary root length of seedlings was also measured under ABA treatment. There were no differences for root growth between controls and transgenic plants under normal growth condition (Fig 3D). Although the exogenous application of ABA (0.5 or 1 μM) inhibited the growth of primary roots in both controls and transgenic lines, the stronger inhibition of root length was observed in control plants compared with transgenic plants (Fig 3E, 3F and 3G). Therefore, *TaPP2C1* overexpression resulted in decreased ABA sensitivity for seed germination and root elongation. Furthermore, expression of ABA biosynthesis (*NtNCED1*), ABA signaling transduction (*NtAREB*), and ABA response (*TobLTP1*) genes were examined in transgenic lines and WT without or with ABA treatment. *NtNCED1*, *NtAREB* and *TobLTP1* showed significantly higher expression in WT than that in OE8 under ABA treatment, while no differences were observed for the expression of these genes in WT and transgenic lines under normal condition (Fig 3H). Collectively, these results indicate that *TaPP2C1* overexpression decreases plant sensitivity to ABA in tobacco.

### Overexpression of *TaPP2C1* improves salt stress resistance in transgenic tobacco

**Fig 4:**
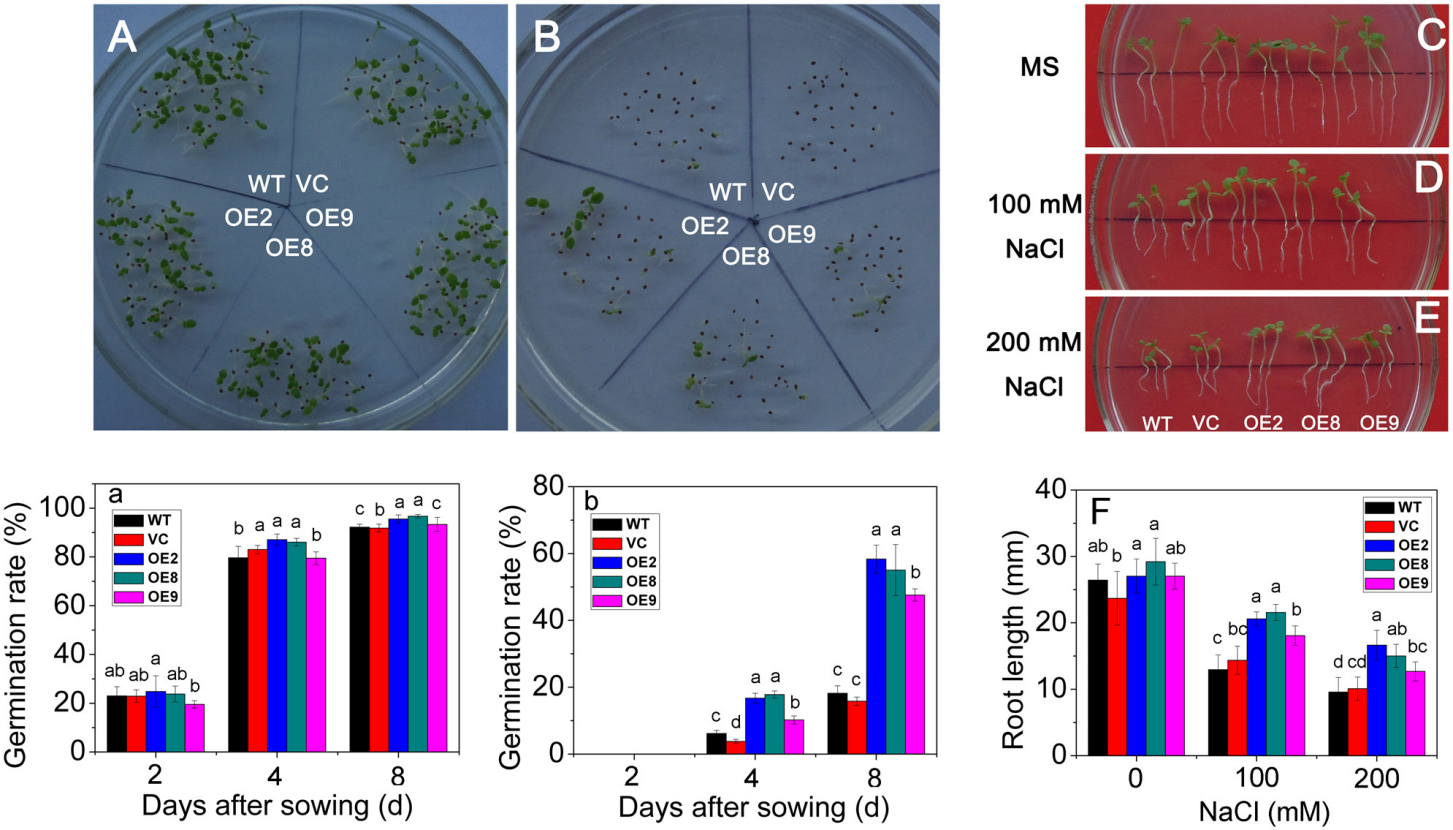
Salt stress tolerance analysis of *TaPP2C1*-overexpressing tobacco plants during seeds germination and root elongation. Seedling phenotypes (A, B) and germination rates (a, b) of WT and transgenic lines under standard condition (A, a) or 200 mM NaCl (B, b) treatment. A and B are photos of the 8 d after germination on media. Seedling phenotypes (C, D, E) and root length (F) of WT and transgenic lines under standard condition (C), 100 mM NaCl (D) or 200 mM NaCl (E) treatment. Data are means ±SD of n = 4 independent experiments. Means denoted by the same letter do not significantly differ at *P* <0.05 as determined by Duncan’s multiple range test.

For salt resistance assay, seeds from control and transgenic plants germinated on MS or MS supplied with 200 mM NaCl were used to detect the germination rates, and results showed that transgenic lines maintained higher germination rates than WT and VC under salt treatment (Fig 4A, 4B, 4a and 4b). In addition, the WT, VC, and transgenic seedlings were transferred to MS or MS supplied with 100–200 mM NaCl for 7 d to detect root length. The result showed that the transgenic lines exhibited less suppression of root growth than control plants under 100–200 mM NaCl treatment (Fig 4D, 4E and 4F), meanwhile there was little difference for root length between WT and the transgenic plants under MS medium (Fig 4C and 4F). Thus, overexpression of *TaPP2C1* improved the adaptation to salt stress during early stages in transgenic tobacco.

**Fig 5:**
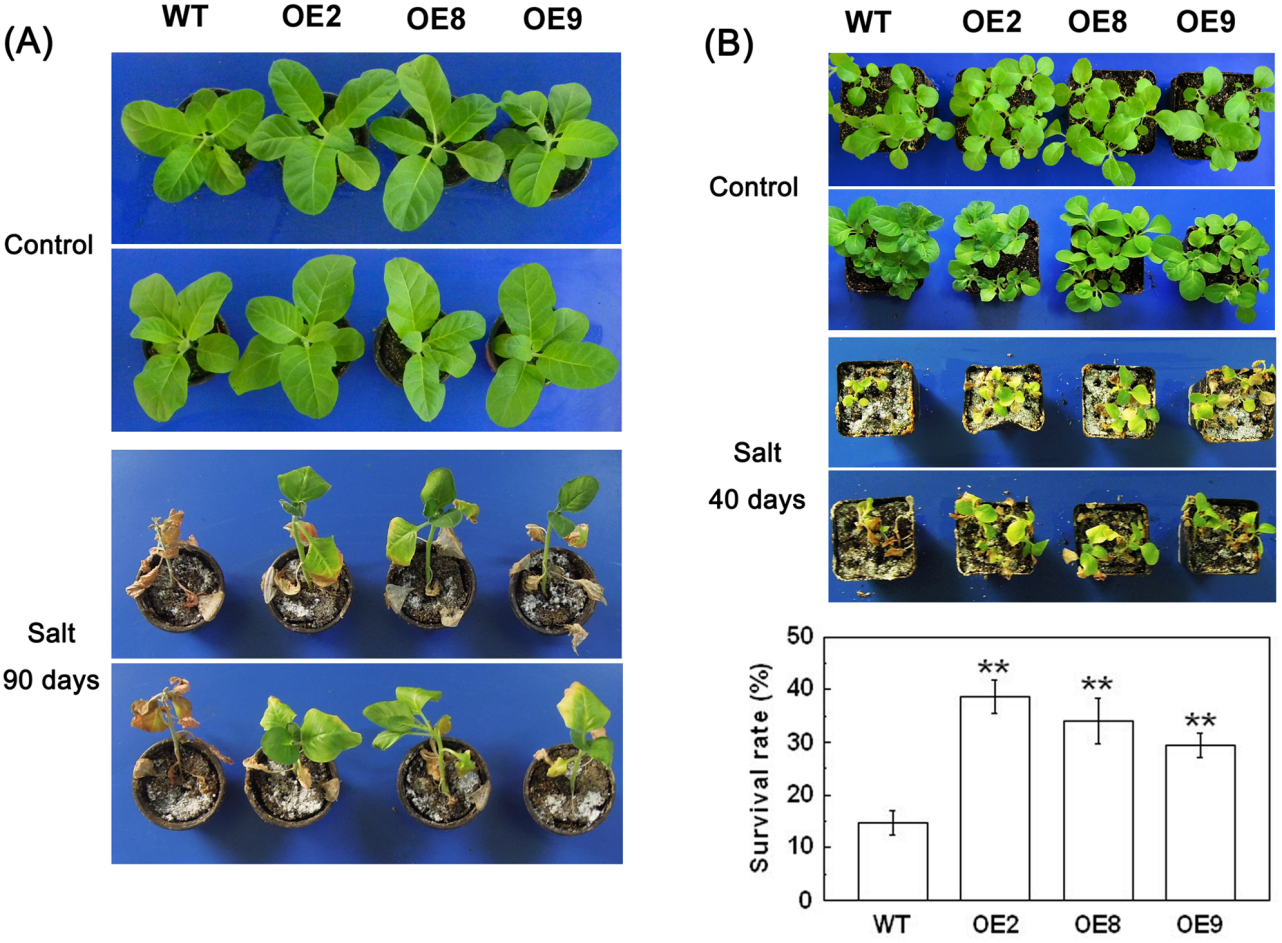
Salt tolerance analysis of *TaPP2C1*-overexpressing tobacco plants at adult stage. (A) Phenotypes of tobacco plants that were grown for 42 d under normal condition and then irrigated with salt solution (300 mM NaCl) for 90 d. (B) Phenotypes and survival rates of tobacco plants that were grown for 21 d under normal condition and then irrigated with salt solution (300 mM NaCl) for 40 d. Data are means ±SD of n = 3 independent experiments. Asterisks indicate significant difference between WT and transgenic lines (*P <0.05; **P <0.01).

In another experiment, 42 d old seedlings grown in pots were irrigated with 300 mM NaCl solution for 90 d. The leaves of the WT plants turned completely yellow, whereas transgenic lines remained alive and displayed some green leaves that turned yellow only in the leaf apex (Fig 5A). Twenty-one days old transgenic and WT plants were also subjected to 40 d treatment with 300 mM NaCl stress. As a result, 14.7% of the WT survived, while the transgenic lines OE2, OE8 and OE9 survived at rates of 38.6, 34.1, and 29.5%, respectively (Fig 5B). These results indicate that *TaPP2C1* overexpression enhances salt stress resistance.

### Overexpression of *TaPP2C1* reduces the content of IL, MDA and H_2_O_2_, and increases the activities of SOD and CAT under salt stress

**Fig 6:**
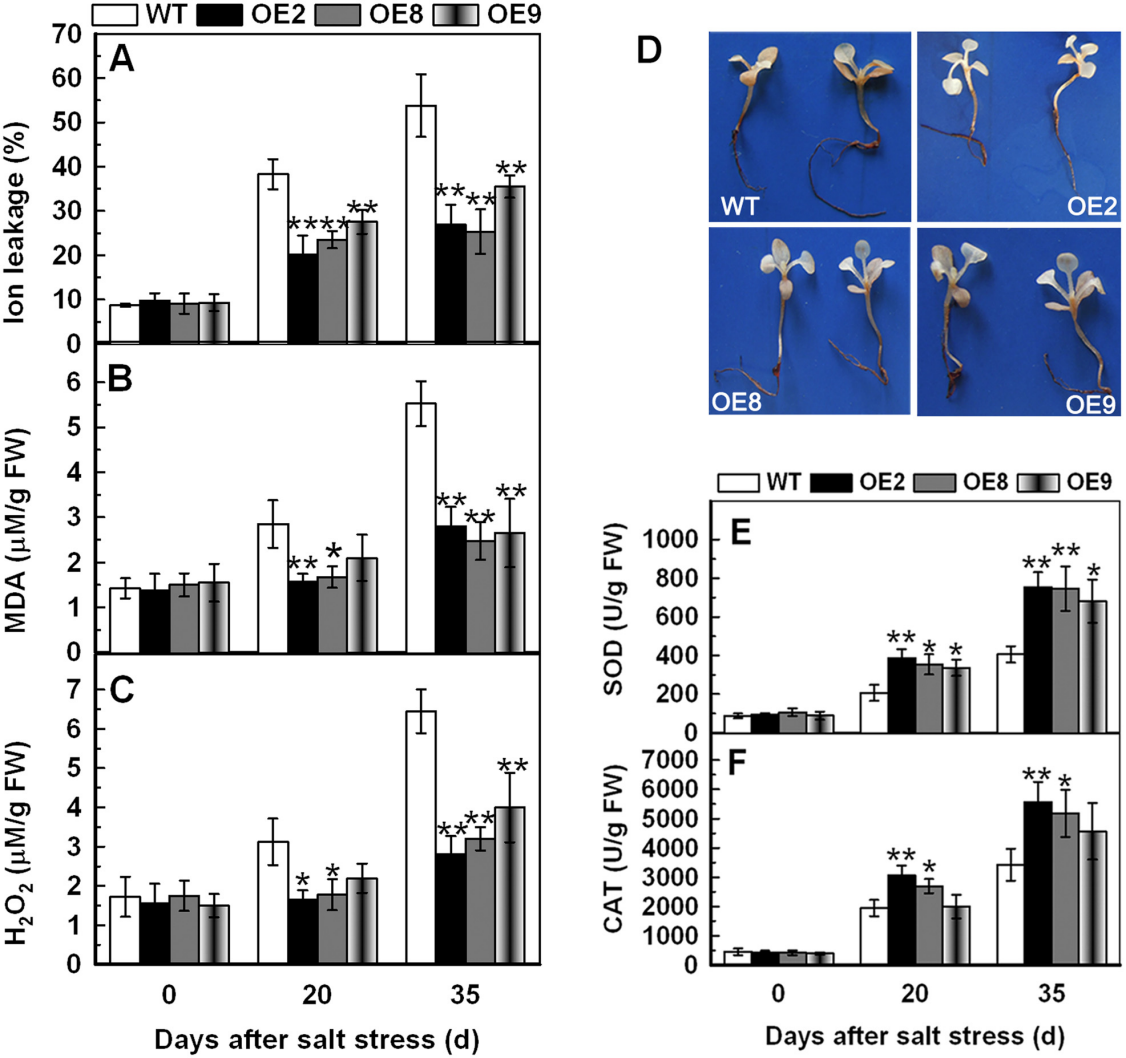
Physiological analysis of WT and transgenic lines under normal and saline condition. Analysis of IL (A), MDA (B) and H_2_O_2_ (C), and the activities of SOD (E) and CAT (F) in the WT and transgenic lines under normal condition and salt treatment. Histochemical detection of H_2_O_2_ accumulation of 7 d old tobacco plants subjected to 7 d 200 mM NaCl stress (D). Data are means ±SD of n = 4 independent experiments. Asterisks indicate significant difference between WT and transgenic lines (*P <0.05; **P <0.01).

IL was obviously lower in transgenic lines than that in WT, indicating that transgenic lines suffered less membrane injury than control plants (Fig 6A). Accordingly, the content of MDA and H_2_O_2_ exhibited similar pattern to IL, significantly lower in transgenic plants relative to the WT (Fig 6B and 6C). In addition, overexpression of *TaPP2C1* resulted in less accumulation of H_2_O_2_ than WT in the root, cotyledon, and shoot apex under salt stress by DAB staining (Fig 6D). These physiological measurements suggest that the transgenic plants are more tolerant to salt stress.

The lower content of IL, MDA and H_2_O_2_ in three transgenic lines compared with the WT under salt treatment implied that they might suffer less membrane injury and lipid peroxidation. Enzymatic antioxidants play key roles in ROS scavenging, thus modulating the cellular ROS levels. Therefore, POD, SOD, and CAT activities were examined in the leaves collected from 21 d old tobacco plants under salt treatment. After 20 and 35 d of salt stress, the transgenic plants showed obviously higher CAT and SOD activities than the WT (Fig 6E and 6F). No significant differences for POD activity were detected between transgenic plants and WT. These results indicat that *TaPP2C1* overexpression increases the activities of CAT and SOD under salt stress.

### *TaPP2C1* regulates the transcription of ABA-associated, ROS scavenging-related, and stress-responsive genes

**Fig 7:**
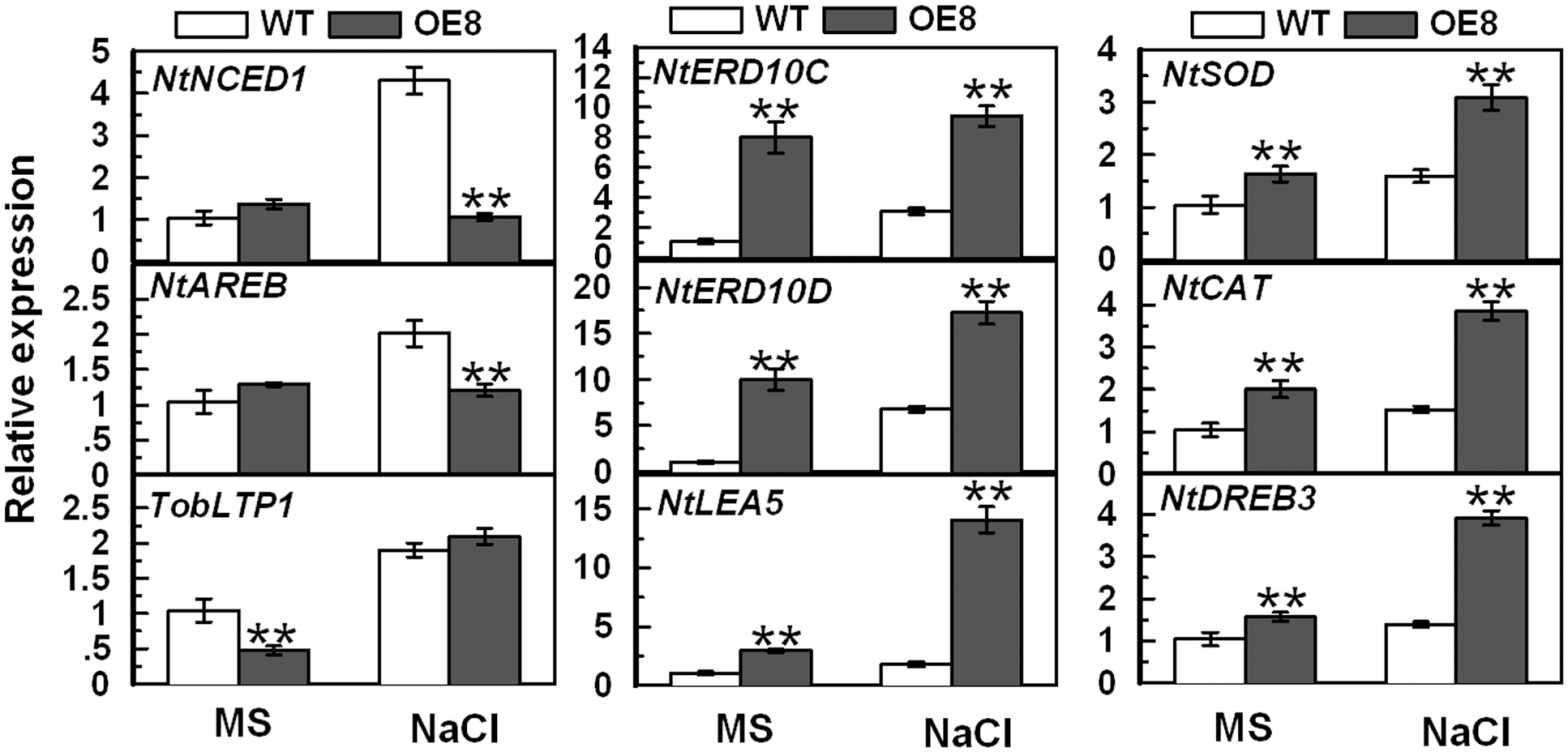
Expression analysis of ABA- and stress- associated genes in the WT and the transgenic line (OE8) under normal condition and salt treatment. The mRNA fold difference is relative to that of WT samples on MS. Data are means ±SD of n = 4 independent experiments. Asterisks indicate significant difference between WT and transgenic lines (*P <0.05; **P <0.01).

To further explore the role of *TaPP2C1* in salt stress response, transcripts of ABA-, stress- and ROS scavenging-related genes were tested under normal condition and salt treatment (Fig 7). The selected genes include ABA biosynthesis (*NtNCED1*), ABA signaling transduction (*NtAREB*), ABA response (*TobLTP1*), stress defense (*NtERD10C*, *NtERD10D* and *NtLEA5*), ROS detoxification (*NtSOD* and *NtCAT*), and regulatory gene (*NtDREB3*). Although the expression of *NtNCED1* and *NtAREB* exhibited no obvious difference between WT and transgenic plants under standard condition, they were significantly higher in WT than in transgenic plants under salt treatment. The expression of *TobLTP1* was higher in WT than that in transgenic plants under standard condition. Notably, the transcripts of *NtERD10C*, *NtERD10D*, *NtLEA5*, *NtSOD*, *NtCAT*, and *NtDREB3* were obviously higher in OE8 than that in WT under normal condition and salt treatment. These results indicate that overexpression of *TaPP2C1* in tobacco decreases the expression of the ABA biosynthesis and ABA signal transduction genes and enhances the ROS-associated and stress-responsive genes under salt treatment.

### Overexpression of *TaPP2C1* increases resistance to oxidative stress in transgenic tobacco

**Fig 8:**
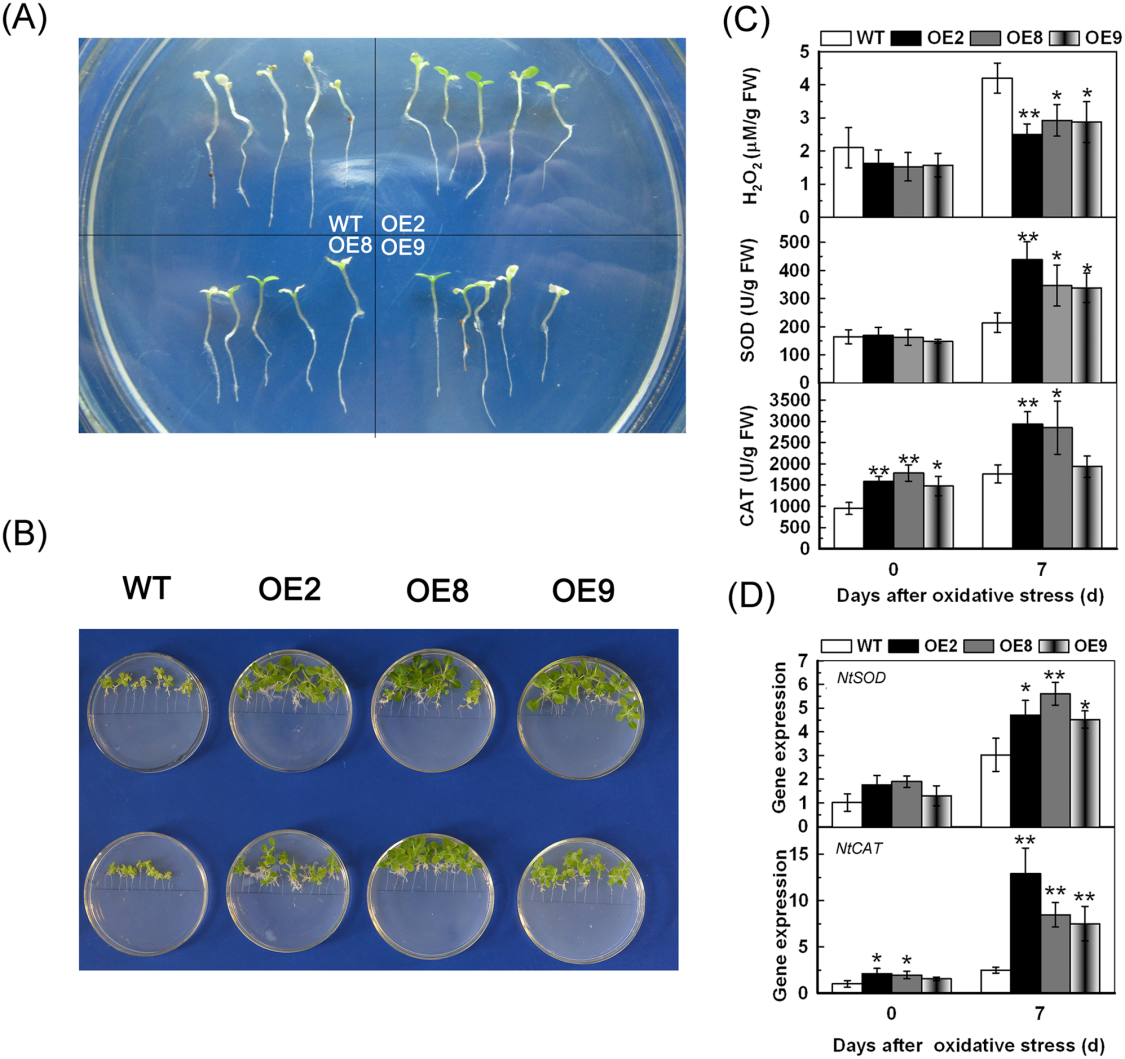
Oxidative stress tolerance analysis of *TaPP2C1*-overexpressing plants. Photos of WT and transgenic lines after MV (A) and H_2_O_2_ (B) treatments. The H_2_O_2_ content and the activities of SOD and CAT in the WT and transgenic lines under normal condition and MV treatment (C). The expression of *NtSOD* and *NtCAT* in the WT and transgenic lines under normal condition and MV treatment (D). Data are means ±SD of n = 4 independent experiments. Asterisks indicate significant difference between WT and transgenic lines (*P <0.05; **P <0.01).

In order to further confirm the involvement of *TaPP2C1* in antioxidant system, the function of *TaPP2C1* in direct oxidative stress was detected. For oxidative stress resistance analysis, 7 d old seedlings were cultured on MS with 30 μM MV for 10 d. Cotyledon bleaching or chlorosis in the WT was more severe than that in the transgenic lines (Fig 8A). In another experiment, 7 d old seedlings were cultured on MS with 5 mM H_2_O_2_ for 14 d. As shown in Fig 8B, the transgenic lines displayed less inhibition of seedlings growth than the WT under H_2_O_2_ treatment. These results suggest that *TaPP2C1* overexpression improves the resistance to oxidative stress in transgenic tobacco.

### Overexpression of *TaPP2C1* reduces H_2_O_2_ accumulation and improves SOD and CAT activities under oxidative stress

In order to examine whether *TaPP2C1* overexpression affects ROS scavenging under oxidative stress, H_2_O_2_ content and activities of SOD and CAT were determined (Fig 8C). The results showed that H_2_O_2_ content and SOD and CAT activities increased in WT and transgenic plants after oxidative stress treatment compared with untreated samples. However, transgenic lines had lower H_2_O_2_ content and higher SOD and CAT activities under oxidative stress compared with WT. We also detected the transcripts of *NtSOD* and *NtCAT*, which showed that transgenic plants exhibited higher transcirption levels of *NtSOD* and *NtCAT* than the WT under oxidative stress (Fig 8D). These results suggested that *TaPP2C1* overexpression reduces the H_2_O_2_ accumulation by improving the activities and expression of SOD and CAT under oxidative stress.

## Discussion

Over the last few decades, research has been focused on clarifying the ABA signaling pathway, among which, group A PP2Cs have been characterized as crucial negative regulators of ABA signaling. However, the role of PP2Cs of other subfamilies remained less known, especially for the group F2 PP2Cs. Genome-wide analyses have identified five genes of group F2 *PP2C* responding to ABA and abiotic stress at transcriptional level in *A*. *thaliana* [4]. Therefore, there is a need to study whether group F2 *PP2C* genes participate in the regulation of ABA signaling and abiotic stress resistance, and whether group F2 *PP2C* genes conferring resistance to abiotic stress is related to ABA signaling. In this study, we characterized the roles of *TaPP2C1*, a group F2 *PP2C* gene, in ABA signaling and salt stress and found: (i) the relationship between *TaPP2C1* and ABA signaling; (ii) the function and physiological mechanism of *TaPP2C1* under salt stress; and (iii) the relationship between *TaPP2C1* function in salt stress and ABA.

### *TaPP2C1* acts as a negative regulator of ABA signaling

In previous studies, PP2C and protein phosphorylation events have been characterized to play crucial roles in ABA signaling pathway in *A*. *thaliana* through identifying the two ABA-insensitive loci ABI1 and ABI2 [22,23]. This concept was further confirmed by evidences demonstrating that double or triple mutants of PP2Cs have increased sensitivity to ABA [2,9,24]. The negative regulatory roles of PP2Cs in ABA signaling were also verified in other plant species [25–27].

In the present study, the transcriptional response of *TaPP2C1* to ABA was investigated in wheat, and it was found that that *TaPP2C1* expression is down-regulated by ABA treatment (Fig 2). Furthermore, *TaPP2C1-*overexpressing tobacco plants displayed ABA insensitivity during seed germination and root elongation (Fig 3). The expression of ABA biosynthesis (*NtNCED1*), ABA signaling transduction (*NtAREB*), and ABA responsive (*TobLTP1*) genes showed lower levels in transgenic plants than in WT with the application of ABA (Fig 3). In addition, *TaPP2C1* overexpression reduced the transcripts of *NtNCED1* and *NtAREB* under salt stress, indicating that the ABA signaling pathway may be inhibited under salt stress in transgenic plants (Fig 7). These results allowed us to conclude that *TaPP2C1* is a negative regulator of ABA signaling.

### *TaPP2C1* confers salt stress resistance by activating the antioxidant system

PP2Cs have been confirmed to play crucial roles in plants responding to abiotic and biotic stress [13,28,29]. Some PP2Cs, such as PP2CA and ABI2, were reported to negatively regulate plant resistance to freezing and salt stresses [12,13], whereas AtPP2CG1 and ZmPP2C2 have been shown to be positive regulators of salt and cold stress resistance [14,30]. In the present study, *TaPP2C1* overexpression enhanced salt stress resistance in 7 d, 14 d, and 42 d old tobacco seedlings (Figs 4 and 5); thus, TaPP2C1 is a positive regulator of salt stress resistance.

As the damaging effects of saline affects various biological processes, such as growth, photosynthesis and metabolism, plants have evolved a series of signaling pathways to relieve the damage of salt stress, including not only ABA signaling but also other plant hormones and signaling molecules. In particular, SA, ethylene, and jasmonic acid have been shown to affect abiotic stress responses through a complex interaction with ABA [31–33]. In addition, PP2Cs can not be modulated by regulatory subunits due to their characteristic of monomeric enzyme. The interactions of substrates or signaling components with PP2Cs are required for PP2Cs obtaining specific function. Thus, PP2C activity might also be affected by protein synthesis, sequestering, or second messengers that include Ca^2+^, H_2_O_2_, and unsaturated fatty acids [34]. Thus, TaPP2C1 may be regulated by ABA, as well as by other multiple signaling molecules supported by the expression patterns of *TaPP2C1* in response to different signaling molecules (Fig 2 and S4 Fig). The obvious induction of *TaPP2C1* after H_2_O_2_ treatment implies a relationship between the improved resistance to salt stress conferred by *TaPP2C1* and H_2_O_2_.

On the basis of physiological analysis, the improved resistance to salt stress in transgenic plants is associated with the maintenance of less IL and MDA content which are important indicators of membrane injury (Fig 6). MDA, the product of lipid peroxidation, is usually used for evaluating ROS-mediated damage in plants [35]. TaPP2C1 overexpression may have been resulted in relieving lipid peroxidation caused by ROS injury under salt treatment. Therefore, there is a need to investigate whether *TaPP2C1* plays a role in ROS scavenging.

The transgenic lines contained lower level of H_2_O_2_ relative to WT under salt stress, implying that scavenging systems of ROS in transgenic plants might be more efficient compared to WT plants (Fig 6). Antioxidant enzymes, such as CAT, SOD and POD play crucial roles in scavenging ROS and protecting the plants from injury caused by abiotic stress [36–38]. Antioxidant enzymes analysis showed that the transgenic plants had obviously higher CAT and SOD activities than WT under salt treatment (Fig 6). Additionally, the higher transcripts of *NtSOD* and *NtCAT* in transgenic plants relative to WT could contribute to the increased activities of NtSOD and NtCAT (Fig 7). These findings suggest that overexpression of the *TaPP2C1* gene improved the antioxidant defense system, and hence protecting transgenic plants from ROS-mediated damage under salt treatment.

To confirm this result, the direct oxidative stress caused by MV and H_2_O_2_ was applied to tobacco plants. The transgenic lines were more tolerant to oxidative stress and had lower H_2_O_2_ content and improved antioxidant system than control plants (Fig 8). The function of PP2C in enhancing cold stress resistance by improving the antioxidative system was identified previously. The *ZmPP2C2*-overexpressing tobacco plants exhibited higher germination rate and activities of SOD, POD and CAT, but lower IL and MDA under cold stress [30]. Here, we provide evidences that *TaPP2C1* confers resistance to salt stress by enhancing activities and expression of SOD and CAT and hence scavenging ROS *in vivo*.

### *TaPP2C1* confers salt stress resistance by inducing transcription of ABA-independent genes

Plants respond to salt stress through the perception and transduction of stress signals, which leads to induction of various stress-responsive genes that is largely regulated by specific transcription factors [1]. Activity of transcription factor can be modulated through protein phosphorylation and dephosphorylation, which results in changes in gene expression and cell behavior [39]. To combat with variable environmental stresses, various transcription factors are involved in both ABA-dependent and ABA-independent signal transduction pathways [40]. ABA-dependent and ABA-independent gene expression systems induced by salt stress mainly include AREB and DREB transcription factors, respectively [1].

In the present study, *TaPP2C1* overexpression decreased the transcripts of ABA biosynthesis and ABA signaling transduction genes, but enhanced DREB transcription factor and LEA class genes expression under salt stress (Fig 7). DREB transcription factors were characterized to regulate a series of target genes, including LEA class proteins [41]. LEA proteins function in stabilizing labile enzymes, binding water and protecting macromolecular structures under abiotic stress [42,43]. *NtERD10 (C/D)* and *NtLEA5* encode group 2 and group 5 LEA proteins, respectively. The induction of these *LEA* genes by *TaPP2C1* overexpression indicate that more LEA proteins might be synthesized in transgenic plants, resulting in less membrane injury and lipid peroxidation, hence reducing the destruction of transgenic plants. Therefore, *TaPP2C1* confers salt stress resistance by activating the ABA-independent signaling pathway.

In conclusion, this study demonstrates that a group F2 *PP2C* gene (*TaPP2C1*) acts as a negative factor of ABA signaling, but as a positive factor of salt stress. *TaPP2C1* confers resistance to salt stress by improving the antioxidant system and ABA-independent gene expression systems. This evidence provides the impetus for further investigation of the exact roles that PP2Cs play in the ABA-independent pathway.

## Supporting Information

S1 Fig Comparison of TaPP2C1 with other known PP2C proteins. Amino acid sequences are aligned by DNAMAN software. Residues hypothetically involved in the coordination of the phosphate and metal ions are marked with stars. The conserved motifs found in PP2C family are indicated in Arabic numbers above each region. The accession numbers of these known proteins in GenBank are as follows: OsPP2C (BAC16709) from Oryza sativa and AtPP2C59 (NP_194903) from Arabidopsis thaliana.
(TIF)

S2 Fig Phylogenetic relationship of TaPP2C1 with PP2Cs of Arabidopsis. The position of TaPP2C1 was marked with box. The numbers beside the branches represent bootstrap values based on 1000 replicates. Phylogenetic tree was constructed by using MEGA 5.0 software.
(TIF)

S3 Fig Relative expression levels of *TaPP2C1* in different wheat tissues by qRT-PCR analysis. R: root; S: stem; L: leaf; ST: stamen; P: pistil; LE: lemma. The mRNA fold difference was relative to that of root samples. Data are means ±SD of n = 4 independent experiments. Means denoted by the same letter do not significantly differ at *P* <0.05 as determined by Duncan’s multiple range test.
(TIF)

S4 Fig Expression profiles of *TaPP2C1* after MeJA, SA, ethylene and auxin treatments in wheat leaves by qRT-PCR analysis. The mRNA fold difference was relative to that of samples at 0 h. Data are means ±SD of n = 4 independent experiments. Means denoted by the same letter do not significantly differ at *P* <0.05 as determined by Duncan’s multiple range test.
(TIF)

S5 Fig Expression of *TaPP2C1* in transgenic tobacco lines. The WT, VC and three transgenic lines were cultured in MS medium for two weeks and the whole seedlings were used to extract RNA to detect gene expression using NtUbiquitin as an internal control. Three independent experiments produced similar results.
(TIF)

S1 Table Primers used for PCR analysis. (DOCX)
