# Supplementary figures and images for: TaPP2C1, a Group F2 Protein Phosphatase 2C Gene, Confers Resistance to Salt Stress in Transgenic Tobacco
Source: PLoS One. 2015 Jun 9;10(6):e0129589. doi: 10.1371/journal.pone.0129589 (PMC4461296; doi:10.1371/journal.pone.0129589)

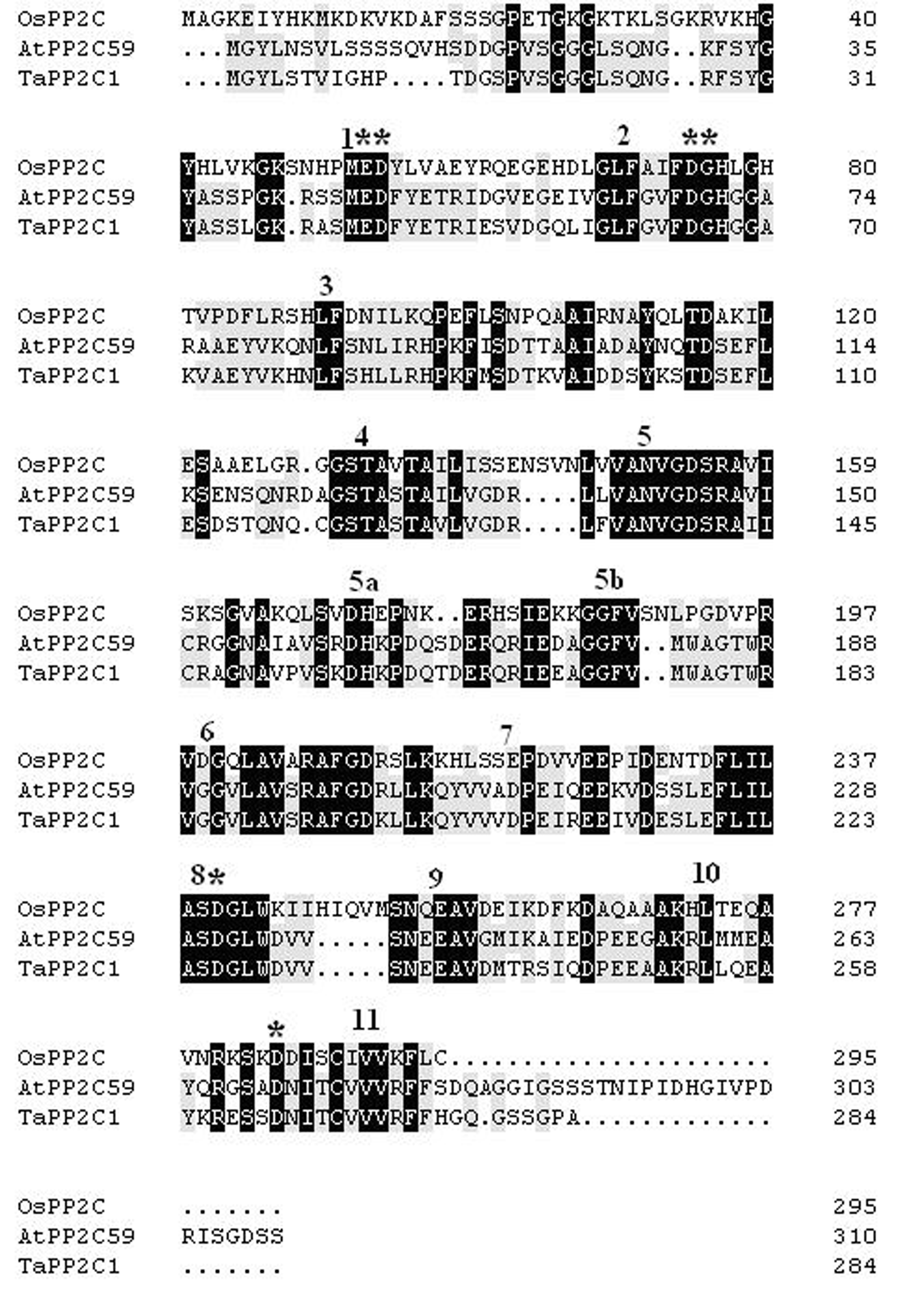

Supplement: S1 Fig — Amino acid sequences are aligned by DNAMAN software. Residues hypothetically involved in the coordination of the phosphate and metal ions are marked with stars. The conserved motifs found in PP2C family are indicated in Arabic numbers above each region. The accession numbers of these known proteins in GenBank are as follows: OsPP2C (BAC16709) from Oryza sativa and AtPP2C59 (NP_194903) from Arabidopsis thaliana. (TIF) [file pone.0129589.s001.tif]

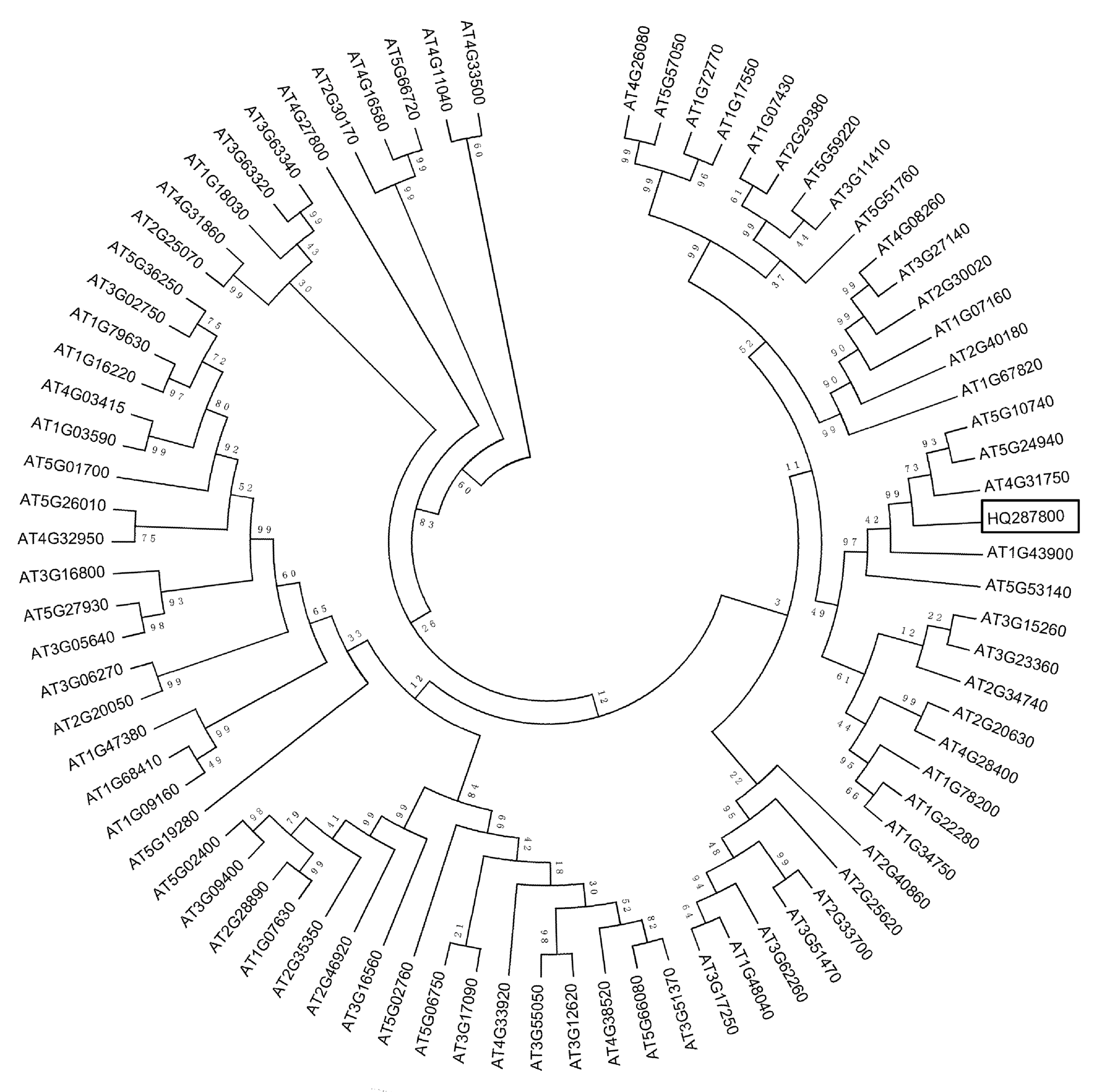

Supplement: S2 Fig — The position of TaPP2C1 was marked with box. The numbers beside the branches represent bootstrap values based on 1000 replicates. Phylogenetic tree was constructed by using MEGA 5.0 software. (TIF) [file pone.0129589.s002.tif]

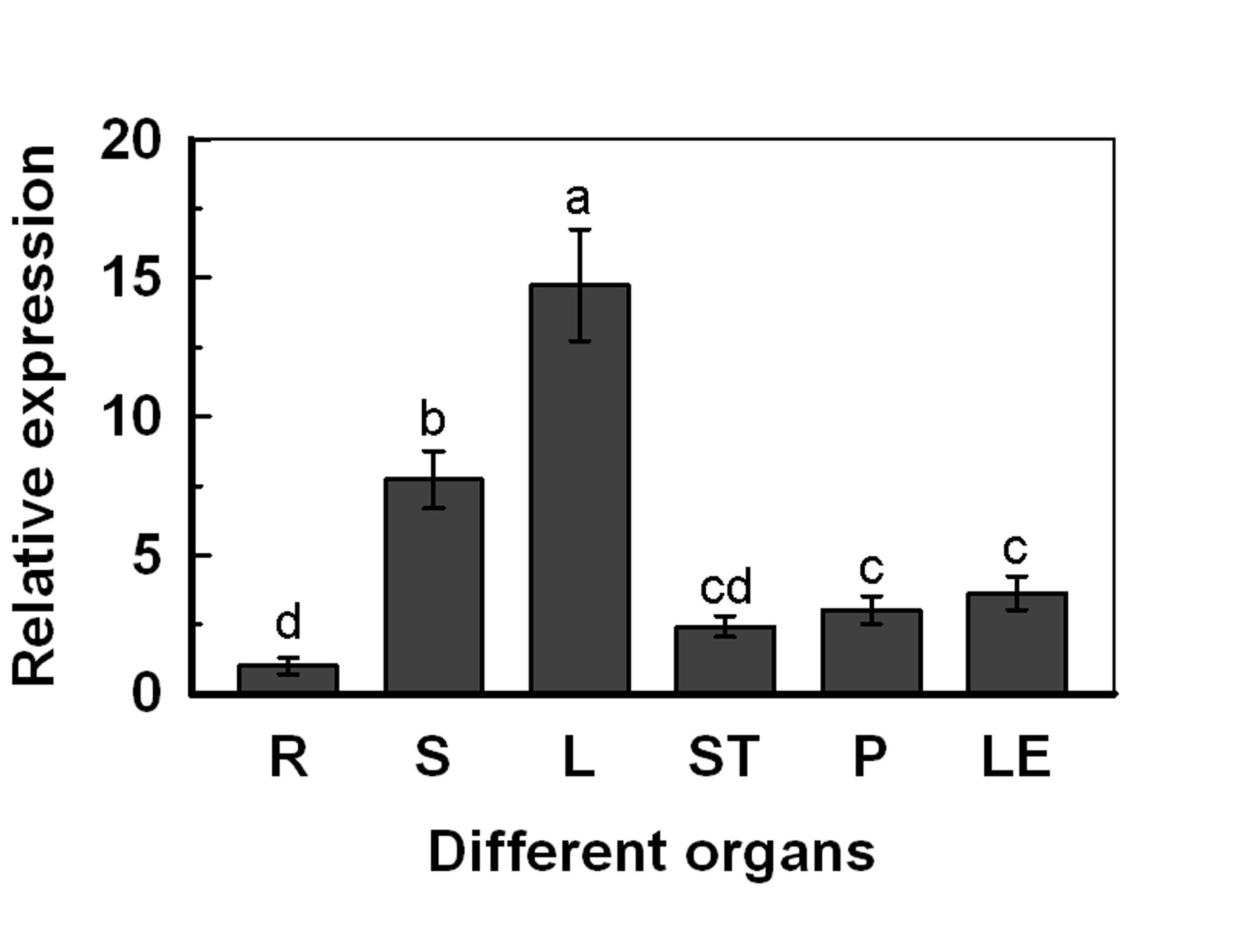

Supplement: S3 Fig — R: root; S: stem; L: leaf; ST: stamen; P: pistil; LE: lemma. The mRNA fold difference was relative to that of root samples. Data are means ±SD of n = 4 independent experiments. Means denoted by the same letter do not significantly differ at P <0.05 as determined by Duncan’s multiple range test. (TIF) [file pone.0129589.s003.tif]

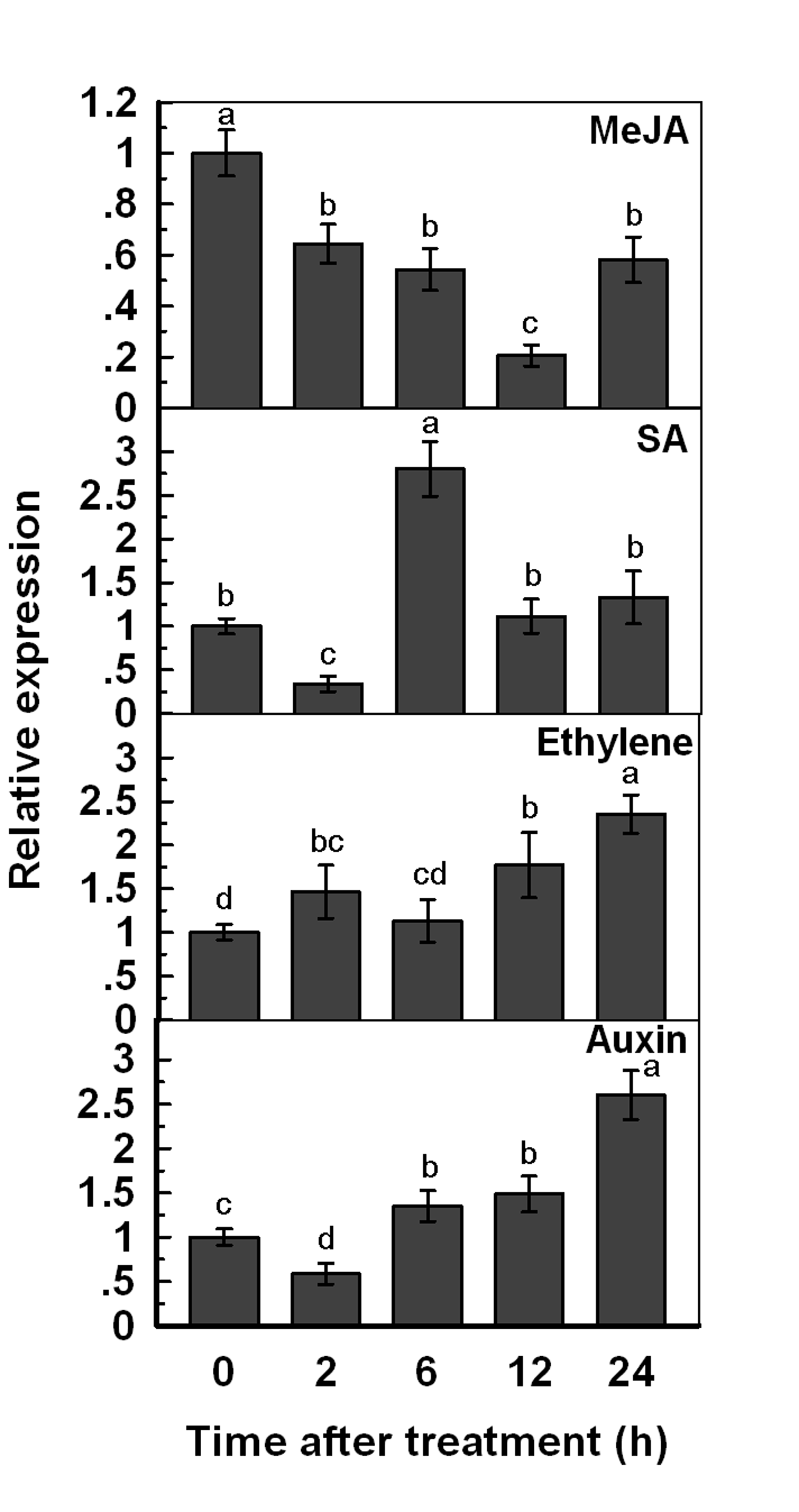

Supplement: S4 Fig — The mRNA fold difference was relative to that of samples at 0 h. Data are means ±SD of n = 4 independent experiments. Means denoted by the same letter do not significantly differ at P <0.05 as determined by Duncan’s multiple range test. (TIF) [file pone.0129589.s004.tif]

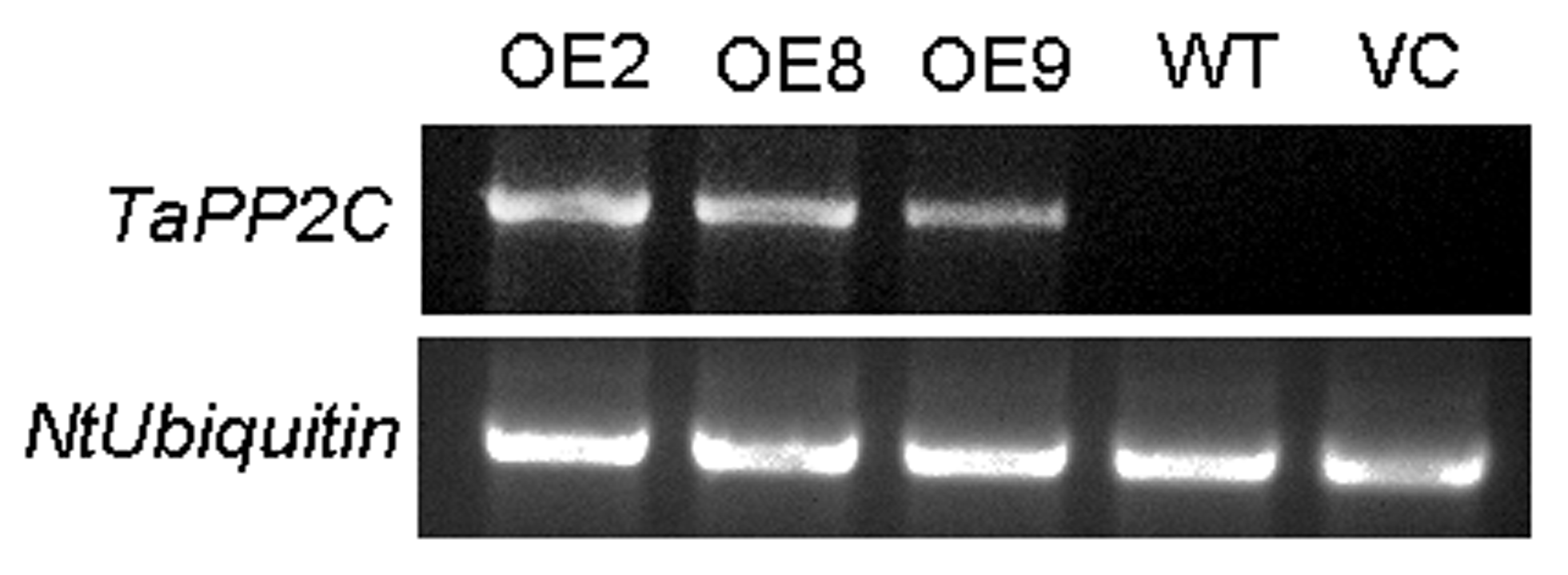

Supplement: S5 Fig — The WT, VC and three transgenic lines were cultured in MS medium for two weeks and the whole seedlings were used to extract RNA to detect gene expression using NtUbiquitin as an internal control. Three independent experiments produced similar results. (TIF) [file pone.0129589.s005.tif]
